# 2-{1-[(6*R*,*S*)-3,5,5,6,8,8-Hexamethyl-5,6,7,8-tetra­hydro­naphthalen-2-yl]ethyl­idene}-*N*-methyl­hydrazinecarbo­thioamide

**DOI:** 10.1107/S2414314623010209

**Published:** 2023-11-30

**Authors:** Ana Paula Lopes de Melo, Alex Fabiani Claro Flores, Leandro Bresolin, Bárbara Tirloni, Adriano Bof de Oliveira

**Affiliations:** a Escola de Química e Alimentos, Universidade Federal do Rio Grande, Av. Itália km 08, Campus Carreiros, 96203-900 Rio Grande-RS, Brazil; b Departamento de Química, Universidade Federal de Santa Maria, Av. Roraima 1000, Campus Universitário, 97105-900 Santa Maria-RS, Brazil; c Departamento de Química, Universidade Federal de Sergipe, Av. Marcelo Deda Chagas s/n, Campus Universitário, 49107-230 São Cristóvão-SE, Brazil; Goethe-Universität Frankfurt, Germany

**Keywords:** thio­semicarbazone, fixolide thio­semicarbazone, chiral thio­semicarbazone, hydrogen-bonded ribbon, fixolide derivative, crystal structure

## Abstract

The reaction between a racemic mixture of (*R*,*S*)-fixolide and 4-methyl­thio­semicarbazide in ethanol with a 1:1 stoichiometric ratio and catalysed with HCl, yielded the title compound, C_20_H_31_N_3_S [common name: (*R*,*S*)-fixolide 4-methyl­thio­semicarbazone]. There is one crystallographically independent mol­ecule in the asymmetric unit, which is disordered over the aliphatic ring [site-occupancy ratio = 0.667 (13):0.333 (13)]. The disorder includes the chiral C atom, the neighbouring methyl­ene group and the methyl H atoms of the methyl group bonded to the chiral C atom. The maximum deviations from the mean plane through the disordered aliphatic ring amount to 0.328 (6) and −0.334 (6) Å [r.m.s.d. = 0.2061 Å], and −0.3677 (12) and 0.3380 (12) Å [r.m.s.d. = 0.2198 Å] for the two different sites. Both fragments show a half-chair conformation. Additionally, the N—N—C(=S)—N entity is approximately planar, with the maximum deviation from the mean plane through the selected atoms being 0.0135 (18) Å [r.m.s.d. = 0.0100 Å]. The mol­ecule is not planar due to the dihedral angle between the thio­semicarbazone entity and the aromatic ring, which amounts to 51.8 (1)°, and due to the *sp* ^3^-hybridized carbon atoms of the fixolide fragment. In the crystal, the mol­ecules are connected by H⋯S inter­actions with graph-set motif *C*(4), forming a mono-periodic hydrogen-bonded ribbon along [100]. The Hirshfeld surface analysis suggests that the major contributions for the crystal cohesion are [(*R*,*S*)-isomers considered separately] H⋯H (75.7%), H⋯S/S⋯H (11.6%), H⋯C/C⋯H (8.3% and H⋯N/N⋯H (4.4% for both of them).

## Abstract 2

The synthesis, crystal structure and Hirshfeld analysis of (*R*,*S*)-fixolide 4-methyl­thio­semicarbazone is reported. The compound is disordered over the fixolide fragment and in the crystal, the mol­ecules are linked by H⋯S inter­actions with graph-set motif *C*(4), forming mono-periodic hydrogen-bonded ribbons along [100].

## Graphical abstract

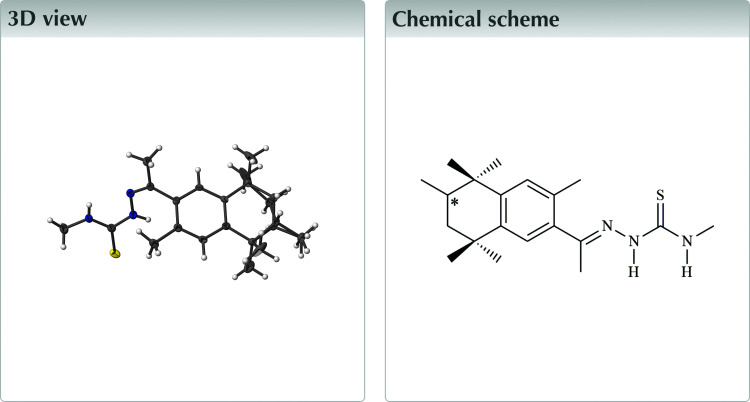

## Structure description

The thio­semicarbazone chemistry is essentially inter­disciplinary and these mol­ecules, characterized by the *R*_1_ *R*_2_N—N(H)—C(=S)—N*R*_3_ *R*_4_ functional group, play an important role in a wide range of scientific disciplines, including biochemistry, coordination chemistry and materials science. Originally, thio­semicarbazone derivatives were the major product of a condensation reaction employed in the organic chemistry for the detection of ketones and aldehydes, using thio­semicarbazide as analytical reagent (Freund & Schander, 1902 ▸). As a result of the huge structural diversity of ketones and aldehydes, a large number of thio­semicarbazone derivatives can be easily obtained for various applications. One of the earliest reports on the application of the thio­semicarbazones can be traced back to the middle of the 1940s, when these compounds were proved to be effective on *Mycobacterium tuberculosis* growth inhibition (Domagk *et al.*, 1946 ▸). Until today, the biological activity of thio­semicarbazone derivatives remains one of the most important approaches for this chemistry. Thio­semicarbazone derivatives are well known for their biological properties, *e.g.*, anti­fungal (Bajaj *et al.*, 2021 ▸), anti­tumoural (Farias *et al.*, 2021 ▸; Rocha *et al.*, 2019 ▸; Siqueira *et al.*, 2019 ▸) and anti-inflammatory pathologies (Kanso *et al.*, 2021 ▸), to cite just a few examples. For instance, thio­semicarbazone coordination compounds also have applications in diagnostic medical imaging and theranostics (Dilworth & Hueting, 2012 ▸; Parrilha *et al.*, 2022 ▸). In addition, thio­semicarbazone complexes are employed as single-mol­ecule precursors in the synthesis of nanostructured materials through thermal decomposition techniques. Thus, Co^II^, Cd^II^ and Zn^II^ complexes are used for the synthesis of CoS and Co_9_S_8_ (Pawar & Garje, 2015 ▸), CdS (Pawar *et al.*, 2016 ▸) and ZnS (Palve & Garje, 2011 ▸) nanoparticles, respectively. For a review of the coordination chemistry of thio­semicarbazones, showing the different bonding modes with diverse metal centres and Lewis acidity, see: Lobana *et al.* (2009 ▸). Finally, thio­semicarbazone derivatives can act as organic corrosion inhibitors, *e.g.*, as a layer of protection for carbon steel AISI 1020 in a hydro­chloric acid medium (Goulart *et al.*, 2013 ▸) and for a theoretical study of the corrosion inhibition concerning dimeric thio­semicarbazones, see: Silva & Martínez-Huitle (2021 ▸).

**Figure 1:**
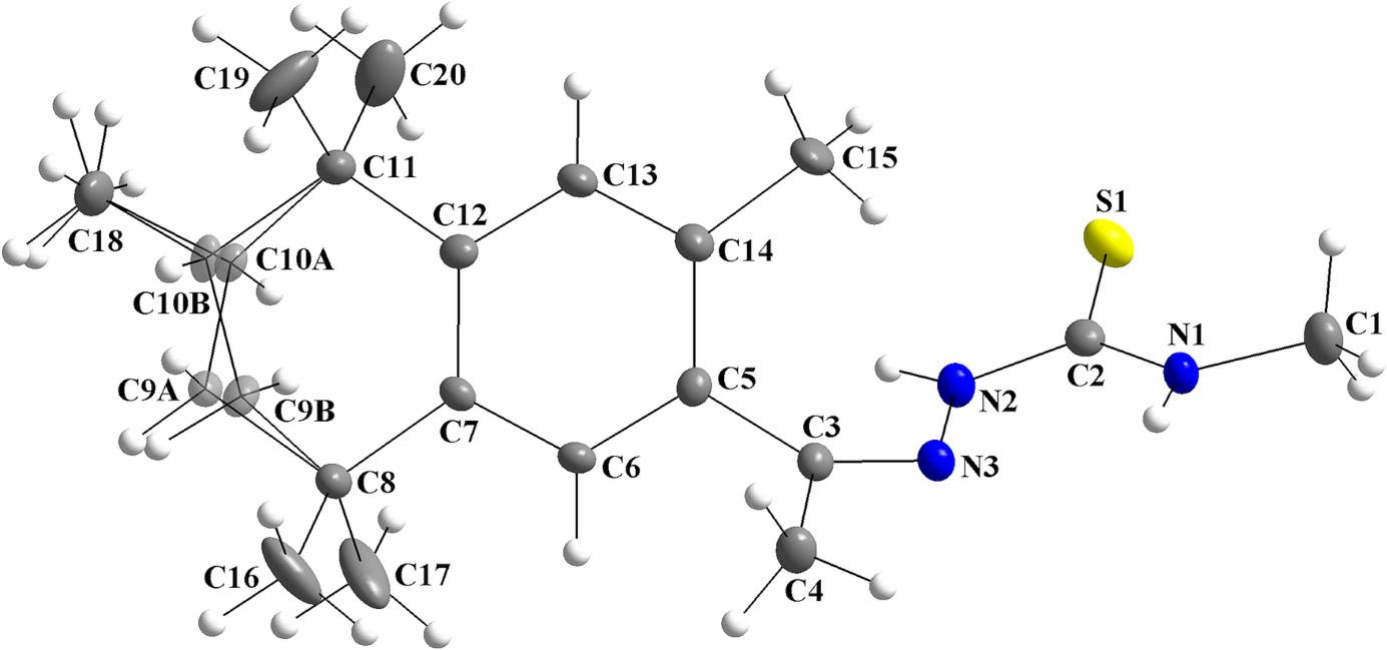
The mol­ecular structure of the title compound, showing the atom labelling and displacement ellipsoids drawn at the 40% probability level. Disordered carbon atoms are drawn with 30% transparency and labelled C9*A*/C10*A* (*R*)-isomer [s.o.f. = 0.667 (13)] and C9*B*/C10*B* for the (*S*)-isomer [s.o.f. = 0.333 (13)].

As part of our inter­est in this chemistry, we report herein the synthesis, crystal structure and Hirshfeld analysis of the title (*R*,*S*)-fixolide 4-methyl­thio­semicarbazone compound. The mol­ecular structure matches the asymmetric unit, which is disordered over the aliphatic ring, with the site-occupancy ratio being 0.667 (13):0.333 (13) for the *A*- and *B*-labelled atoms, respectively (Fig. 1 ▸). A racemic mixture of fixolide was employed as starting material. As the disorder includes the C10 chiral centre, with C10*A*—H10*A* and C10*B*—H*B* bonds in opposite directions, (*R)*- and (*S)*-isomers are observed. The C9 atom was also split over two positions into C9*A* and C9*B*, with the same respective occupancy ratio. The C18 atom is itself not disordered, but the H atoms attached to the carbon atom of this methyl group were refined as disordered to get the best orientations for the C—H bonds, since it is bonded to the *sp* ^3^-hybridized C10*A* and C10*B* atoms. The displacement ellipsoids for C16, C17, C19 and C20 are prolate-like, but no disorder was suggested by the data analysis.

The maximum deviations from the mean plane through the C7/C8/C9*A*/C10*A*/C11/C12 atoms amounts to 0.328 (6) Å for C9*A* and −0.334 (6) Å for C10*A* (r.m.s.d. = 0.2061 Å). The torsion angle for the C8/C9*A*/C10*A*/C11 atom chain is −65.3 (7)° and the aliphatic ring adopts a half-chair conformation. Considering the C7/C8/C9*B*/C10*B*/C11/C12 entity, the deviations amount to −0.3677 (12) Å for C9*B* and 0.3380 (12) Å for C10*B* (r.m.s.d. = 0.2198 Å) and the torsion angle for the C8/C9*B*/C10*B*/C11 chain is 70.2 (14)°, which also resembles a half-chair conformation for the ring.

Concerning the thio­semicarbazone entity, the torsion angles for the N3/N2/C2/N1 and the N3/N2/C2/S1 atom chains amount to 1.2 (4) and −178.1 (2)°, respectively. The maximum deviation from the mean plane through the N3/N2/C2/S1/N1 atoms is 0.0135 (18) Å for N2 (r.m.s.d. = 0.0100 Å), thus, the fragment is approximately planar. The mol­ecule of the title compound is not planar due to the *sp* ^3^-hybridized C atoms of the apliphatic ring and due to the dihedral angle between the mean plane through the N3/N2/C2/S1/N1 atoms and the mean plane through the aromatic ring of the fixolide fragment, which amounts to 51.8 (1)°.

**Figure 2:**
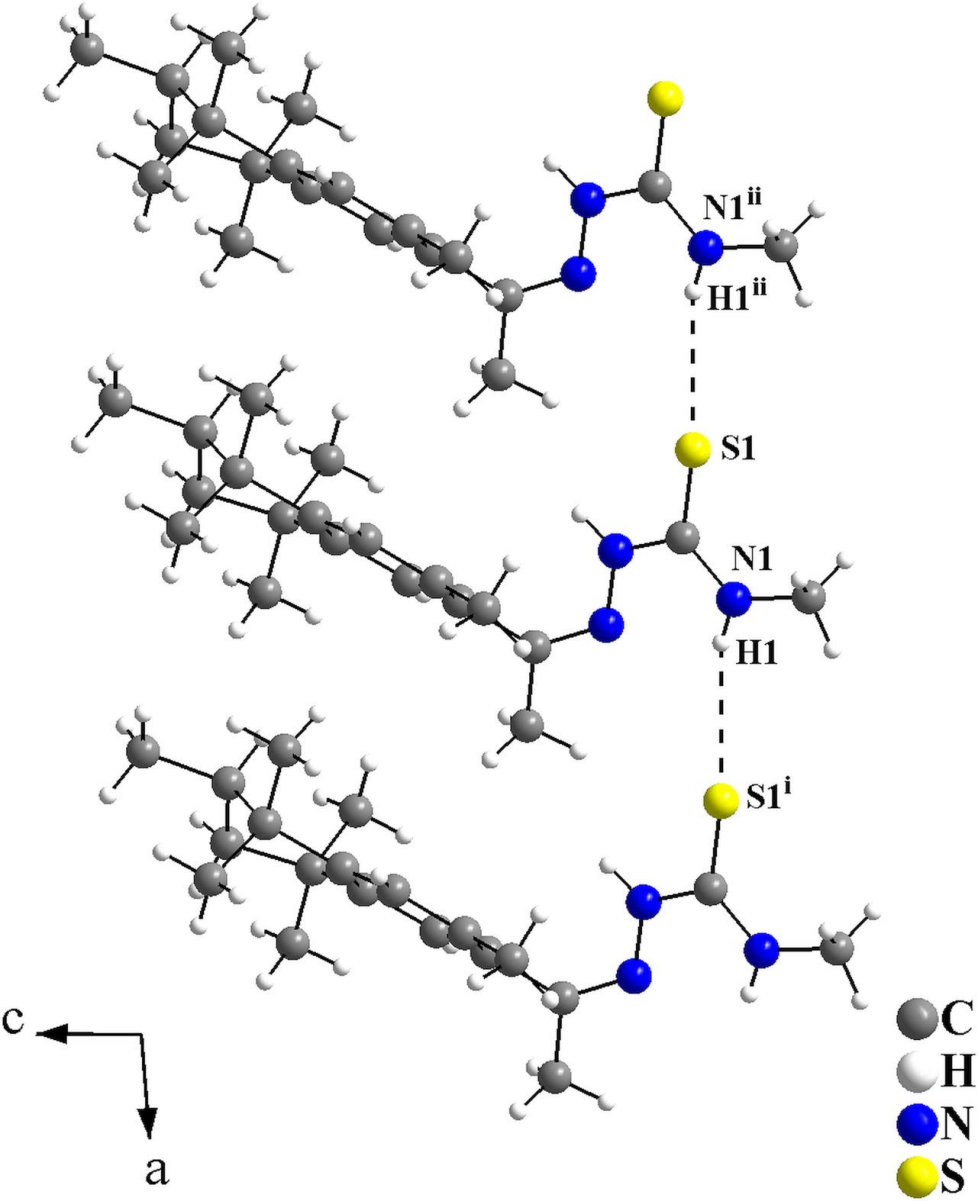
Graphical representation of the H⋯S inter­molecular inter­actions for the title compound viewed along [010]. The inter­actions are drawn as dashed lines, with graph-set motif *C*(4), and connect the mol­ecules into a mono-periodic hydrogen-bonded ribbon along [100]. Only the major occupied sites are drawn for clarity. [Symmetry codes: (i) *x* + 1, *y*, *z*; (ii) *x* − 1, *y*, *z*.]

**Figure 3:**
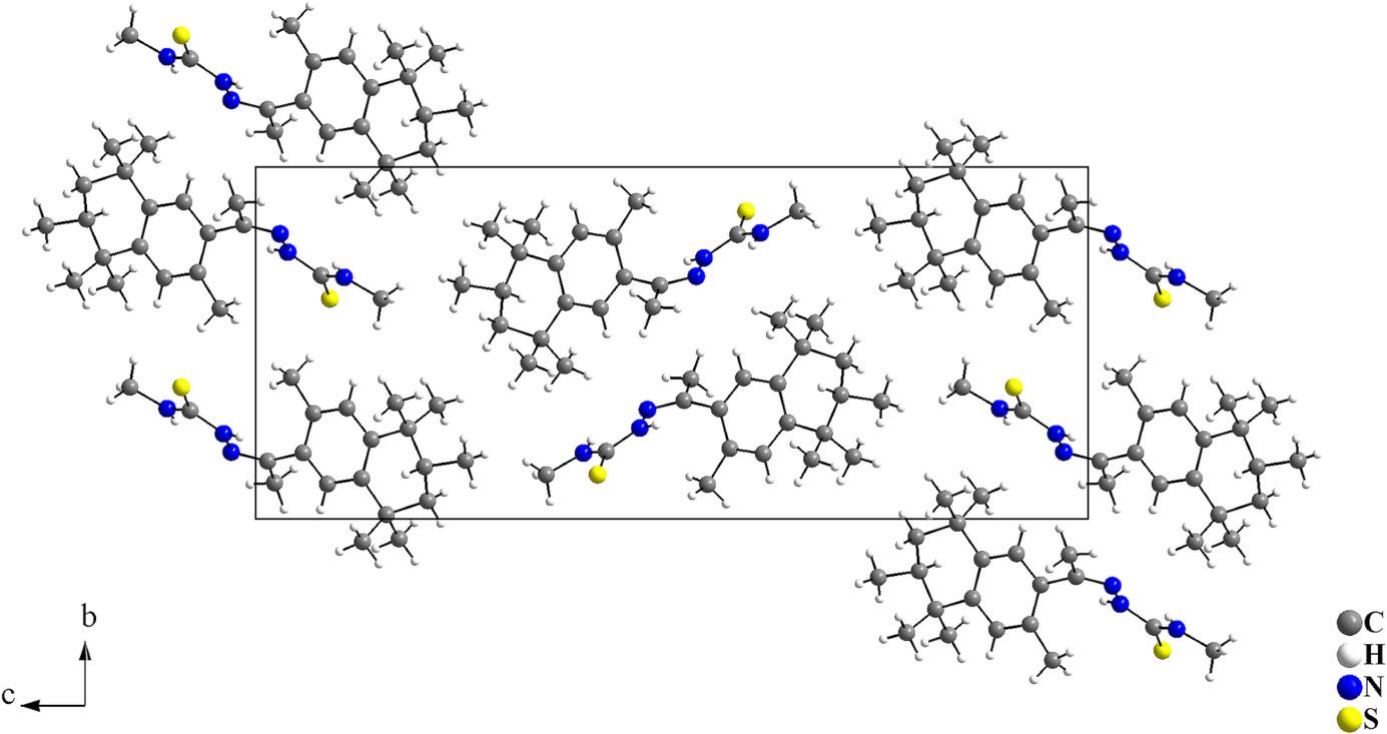
Section of the crystal packing of the title compound. The arrangement of the mol­ecules shows a zigzag or a herringbone motif when viewed along [100]. Only the major occupied sites are drawn for clarity.

In the crystal, the mol­ecules are connected by N—H⋯S inter­actions, with graph-set motif *C*(4), forming a mono-periodic hydrogen-bonded ribbon along [100] (Fig. 2 ▸, Table 1 ▸). The mol­ecular arrangement resembles a zigzag or a herringbone motif when viewed along [100] (Fig. 3 ▸).

**Figure 4:**
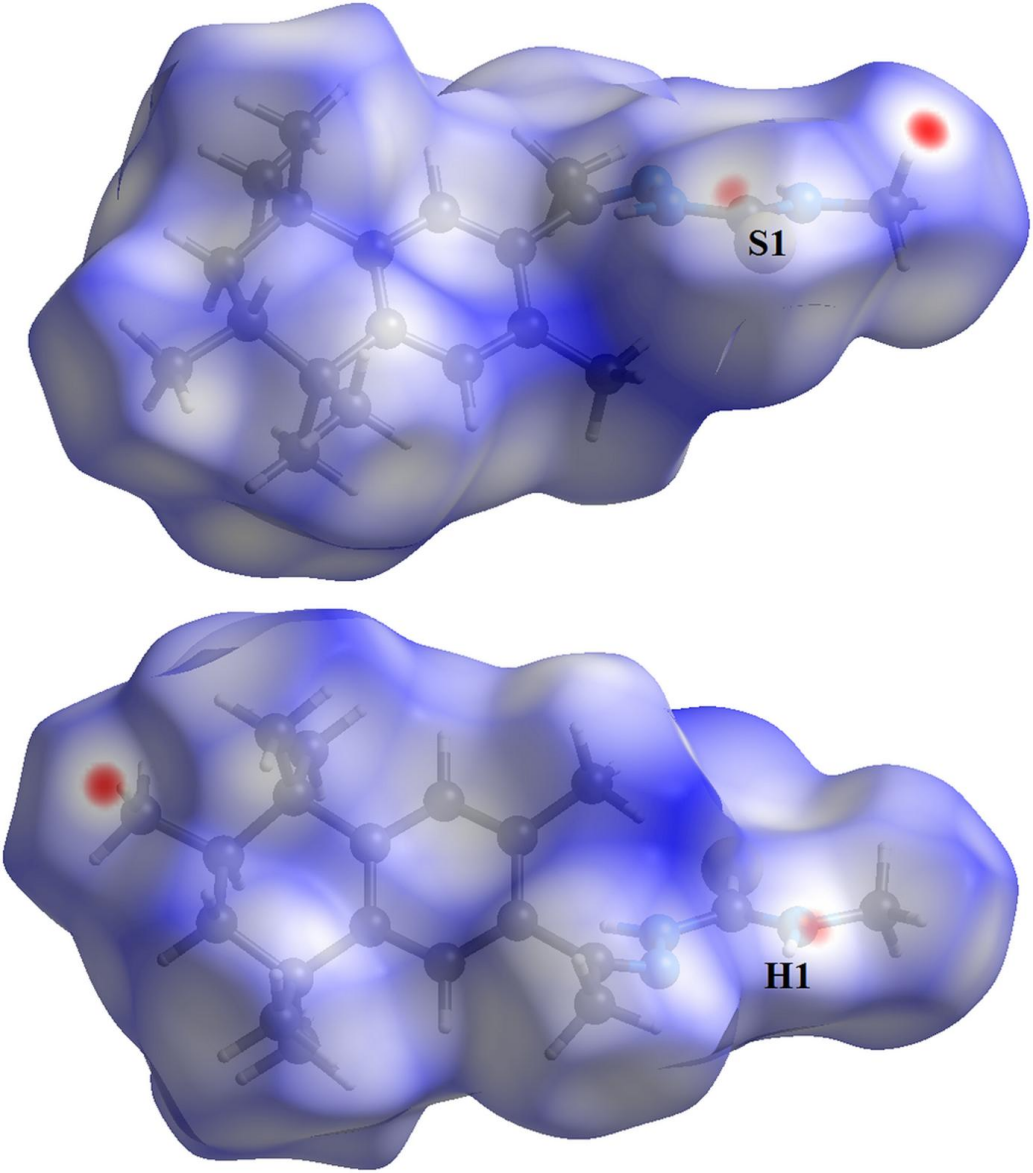
Two opposite side-views in separate figures of the Hirshfeld surface graphical representation (*d*_norm_) for the title compound. The surface is drawn with transparency and simplified for clarity. The regions with strongest inter­molecular inter­actions are shown in red. (*d*_norm_ range: −0.142 to 1.510.)

The Hirshfeld surface analysis (Hirshfeld, 1977 ▸), the graphical representations and the two-dimensional Hirshfeld surface fingerprint plots for the title compound were performed using *CrystalExplorer* (Wolff *et al.*, 2012 ▸). The Hirshfeld surface analysis of the crystal structure indicates that the most relevant inter­molecular inter­actions for crystal cohesion are H⋯H (75.7%), H⋯S/S⋯H (11.6%), H⋯C/C⋯H (8.3% and H⋯N/N⋯H (4.4%). The graphics of the Hirshfeld surface of the title compound are represented with transparency in two opposite side-views and separate figures for clarity (Fig. 4 ▸). The locations of the strongest inter­molecular contacts are indicated in red, *i.e*, the regions around the H1 and S1 atoms. These atoms are those involved in the H⋯S inter­actions shown in a previous figure (Fig. 2 ▸) and in Table 1 ▸.

Although the Hirshfeld surface graphical representation shows, in red, locations of inter­molecular contacts involving H atoms attached to C atoms, no C—H⋯H—C inter­molecular inter­actions can be assigned. The fixolide fragment is a non-polar organic periphery and only weak inter­molecular inter­actions, *e.g.*, London dispersion forces, can be considered. The contribution of H⋯H inter­molecular inter­actions in the supra­molecular arrangement of crystal structures has been studied (Almeida *et al.*, 2017 ▸), but this is not the focus of the present work. The crystal structure of the title compound is disordered, the H atoms were placed geometrically, the *R-*factor amounts to 0.079 and no additional experiment for the inter­molecular inter­actions was performed, so it is not recommended to assure such contacts here. Additionally, no short H⋯H inter­molecular distances were observed.

**Figure 5:**
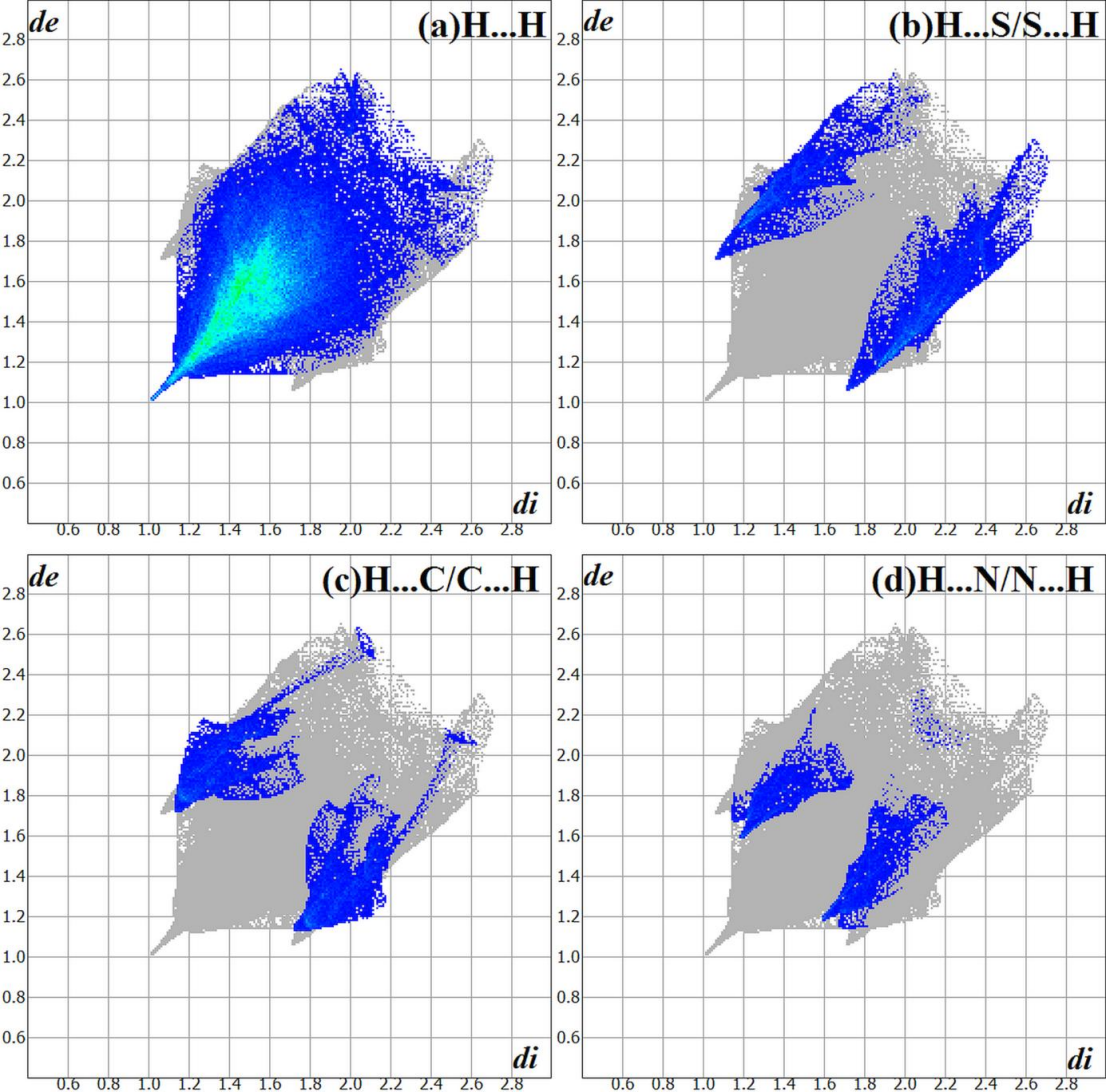
The Hirshfeld surface two-dimensional fingerprint plot for the the title compound showing the (*a*) H⋯H (75.7%), (*b*) H⋯S/S⋯H (11.6%), (*c*) H⋯C/C⋯H (8.3%) and (*d*) H⋯N/N⋯H (4.4%) contacts in detail (cyan dots) and the contributions of the inter­actions for the crystal packing. The *d_i_* (*x-*axis) and the *d_e_* (*y-*axis) values are the closest inter­nal and external distances from given points on the Hirshfeld surface (in Å).

The contributions to the crystal packing are shown as two-dimensional Hirshfeld surface fingerprint plots with cyan dots (Fig. 5 ▸). The *d_i_* (*x-*axis) and the *d_e_* (*y-*axis) values are the closest inter­nal and external distances from given points on the Hirshfeld surface (in Å).

To the best of our knowledge and from using database tools such as *SciFinder* (Chemical Abstracts Service, 2023 ▸) and the Cambridge Structural Database (CSD, accessed *via* WebCSD on November 18, 2023; Groom *et al.*, 2016 ▸), this work represents the first report on the synthesis, crystal structure and Hirshfeld analysis of the fixolide 4-methyl­thio­semicarbazone mol­ecule. Thus, two crystal structures with similarities to the title compound were selected for comparison.

**Figure 6:**
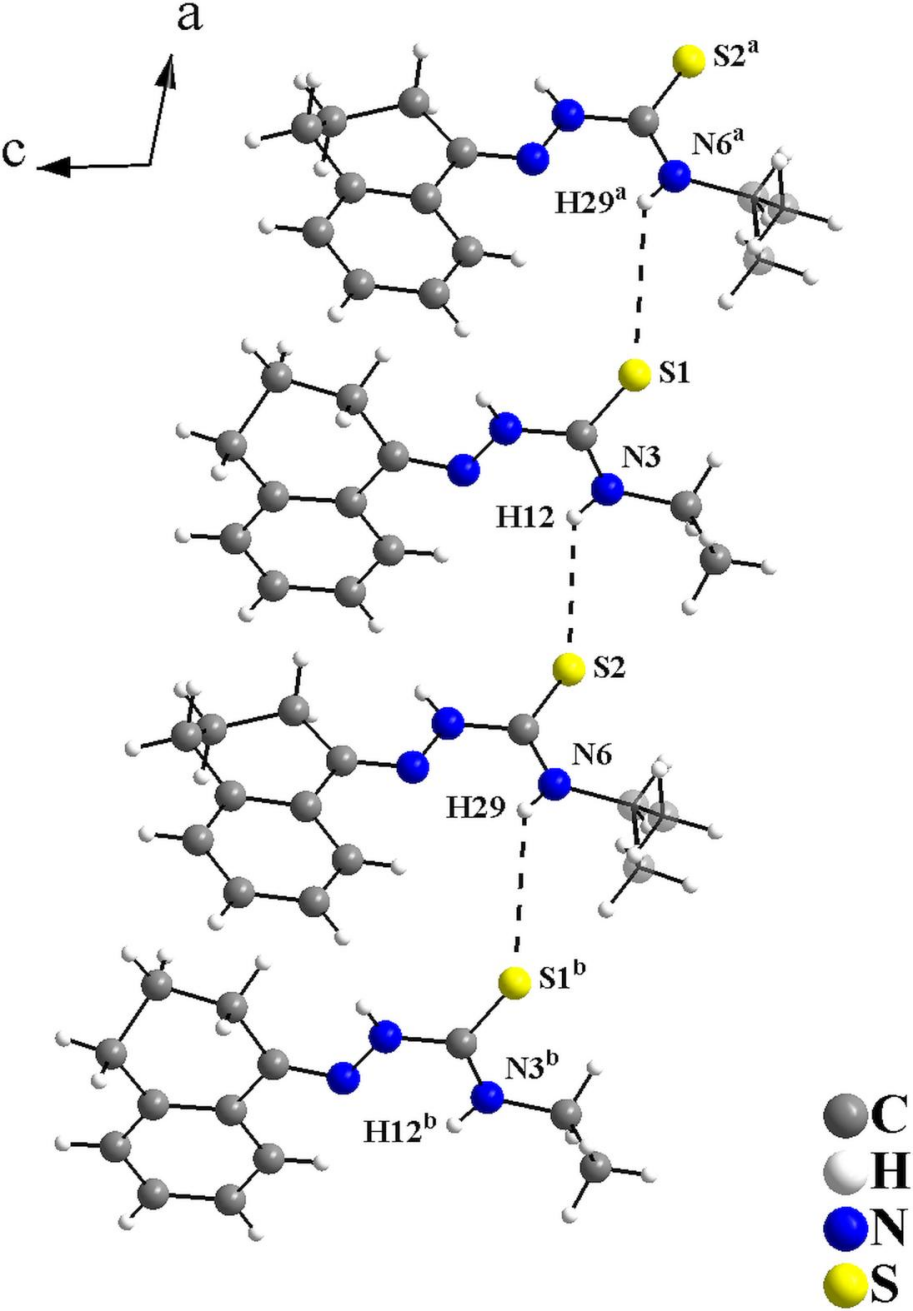
Graphical representation of the H⋯S inter­molecular inter­actions for the tetra­lone 4-ethyl­thio­semicarbazone structure (Oliveira *et al.*, 2017 ▸) viewed along [0 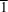 0]. The inter­actions are drawn as dashed lines and link the mol­ecules along [100] with graph-set motif *C*(4), forming a mono-periodic hydrogen-bonded ribbon. Disordered atoms are drawn with 40% transparency. [Symmetry codes: (*a*) *x* + 1, *y*, *z*; (*b*) *x* − 1, *y*, *z*.]

The first selected example is the crystal structure of the tetra­lone 4-ethyl­thio­semicarbazone (Oliveira *et al.*, 2017 ▸). There are two mol­ecules with atoms in general positions forming the asymmetric unit, one of them being disordered over the ethyl fragment. In the crystal, the mol­ecules are linked by H⋯S inter­actions along [100], with graph-set motif *C*(4), and forming a mono-periodic hydrogen-bonded ribbon (Fig. 6 ▸), as observed to the title compound (Fig. 2 ▸). The tetra­lone entity consists of one aliphatic and one aromatic ring, and for the non-polar organic periphery are suggested weak inter­molecular inter­actions only, since even π–π inter­actions are not present in the structure.

**Figure 7:**
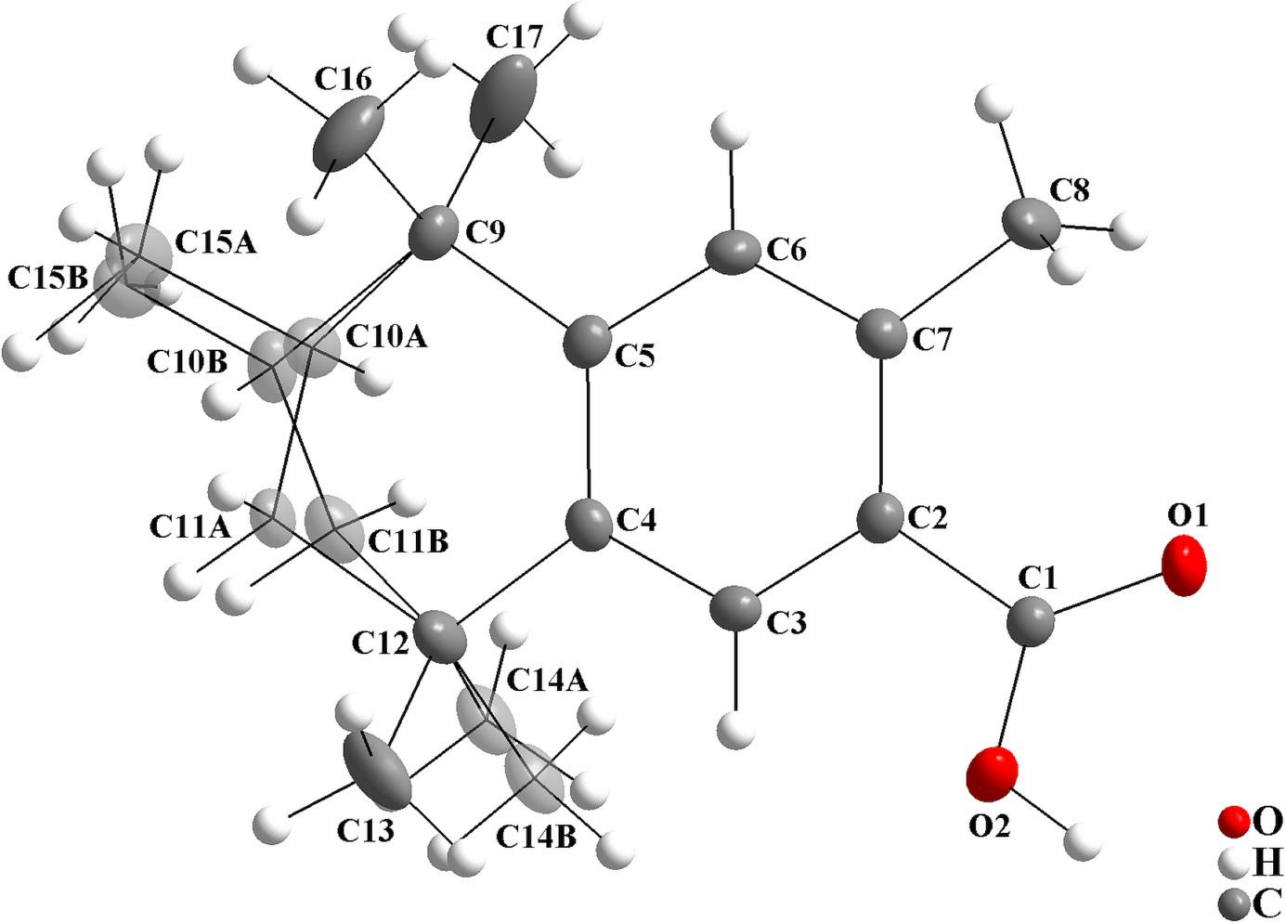
The mol­ecular structure of the (*R*,*S*)-fixolide carb­oxy­lic acid derivative, showing the atom labelling and displacement ellipsoids drawn at the 40% probability level (Kuhlich *et al.*, 2010 ▸). Disordered atoms are drawn with 40% transparency and labelled C10*A*, C11*A*, C14*A* and C15*A* for the (*R*)-isomer [s.o.f. = 0.683 (4)] and C10*B*, C11*B*, C14*B* and C15*B* for the (*S*)-isomer [s.o.f. = 0.317 (4)].

The second example is the crystal structure of a (*R*,*S*)-fixolide carb­oxy­lic acid derivative (Kuhlich *et al.*, 2010 ▸). For this structure, only one crystallographic independent mol­ecule is observed in the asymmetric unit, which shows disorder over the aliphatic ring and two methyl groups (Fig. 7 ▸). The chiral centre is disordered, C10*A* and C10*B*, and two isomers are observed, namely the (*R*)- and (*S*)-forms. For the synthesis, a racemic mixture of fixolide was used as starting material. For the (*R*,*S*)-fixolide carb­oxy­lic acid derivative, the s.o.f. ratio amounts to 0.683 (4):0.317 (4). The torsion angles of the C9/C10*A*/C11*A*/C12 and the C9/C10*B*/C11*B*/C12 atom chains amount to −67.0 (3) and 71.8 (6)°, respectively, being similar to the selected chains of the title compound (Table 2 ▸).

## Synthesis and crystallization

The starting materials were commercially available and were used without further purification. The synthesis of the title compound was adapted from previously reported procedures (Freund & Schander, 1902 ▸; Oliveira *et al.*, 2017 ▸). A mixture of the racemic fixolide (5 mmol) and 4-methyl­thio­semicarbazide (5 mmol) in ethanol (80 ml), catalysed with HCl, was stirred and refluxed for 8 h. After cooling at room temperature, a colourless crystalline solid precipitated, was filtered off and washed with cold ethanol. The crystalline solid was dissolved in warm ethanol and single crystals suitable for X-ray diffraction were obtained by slow evaporation of the solvent at room temperature. The site-occupancy ratio of the disordered atoms refined to 0.667 (13):0.333 (13).

## Refinement

Crystal data, data collection and structure refinement details are summarized in Table 3 ▸. The crystallographically independent mol­ecule is disordered over the aliphatic ring (C9*A*, C9*B*, C10*A* and C10*B*) (Fig. 1 ▸). The s.o.f. for the *A*-labelled atoms amounts to 0.667 (13), while for the *B*-labelled atoms it is 0.333 (13). Although the displacement ellipsoids of C16, C17, C19 and C20 are prolate-like in comparison with the ellipsoids of other methyl groups, *e.g.*, C1, C4, C15 and C18, no additional disorder was indicated by the data analysis.

The hydrogen atoms attached to carbon and nitro­gen atoms were positioned with idealized geometry and constrained to ride on their parent atoms. To get the best orientations for the C—H bonds of the C18H_3_ group, which is bonded to the C10*A* and C10*B* atoms, the methyl hydrogen atoms were split into two positions, located geometrically and refined using a riding model [*U*_iso_(H) = 1.5*U*_eq_(C); C—H bonds lengths set to 0.96 Å]. The other methyl groups were allowed to rotate but not to tip to best fit the experimental electron density and the same C—H bond lengths value was set, also with *U*_iso_(H) = 1.5*U*_eq_(C). The *U*_iso_(H) = 1.2*U*_eq_(C) relation was employed for the other C—H bonds and, for the phenyl ring H atoms, the C—H bond lengths were set to 0.93 Å. For the disordered –CH_2_– fragment (C9*A* and C9*B*), the C—H bond-length value was set to 0.97 Å and for the disordered tertiary C atoms (C10*A* and C10*B*), the C—H bond lengths amount to 0.98 Å. Finally, the N—H bond lengths, with *U*_iso_(H) = 1.2*U*_eq_(N), were set to 0.86 Å.

## Supplementary Material

Crystal structure: contains datablock(s) I. DOI: 10.1107/S2414314623010209/bt4144sup1.cif

Structure factors: contains datablock(s) I. DOI: 10.1107/S2414314623010209/bt4144Isup2.hkl

CCDC reference: 2302507

Additional supporting information:  crystallographic information; 3D view; checkCIF report

## Figures and Tables

**Table 1:** Hydrogen-bond geometry (Å, °)

*D*—H⋯*A*	*D*—H	H⋯*A*	*D*⋯*A*	*D*—H⋯*A*
N1—H1⋯S1^i^	0.86	2.87	3.445 (3)	126

Symmetry code: (i) 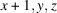 .

**Table 2:** Selected torsion angles (°) for the disordered fixolide 4-methyl­thio­semicarbazone and the fixolide carb­oxy­lic acid derivatives

Compound	Isomer	Chiral atom (s.o.f.)	Atom chain	Torsion angle
C_20_H_31_N_3_S^*a*^	*R*	C10*A* [0.667 (13)]	C8—C9*A*—C10*A*—C11	−65.3 (7)
C_20_H_31_N_3_S^*a*^	*S*	C10*B* [0.333 (13)]	C8—C9*B*—C10*B*—C11	70.2 (14)
C_17_H_24_O_2_^*b*^	*R*	C10*A* [0.683 (4)]	C9—C10*A*—C11*A*—C12	−67.0 (3)
C_17_H_24_O_2_^*b*^	*S*	C10*B* [0.317 (4)]	C9—C10*B*—C11*B*—C12	71.8 (6)

Notes: (*a*) (*R*,*S*)-Fixolide 4-methyl­thio­semicarbazone, reported in this work (Fig. 1 ▸); (*b*) (*R*,*S*)-fixolide carb­oxy­lic acid derivative (Kuhlich *et al.*, 2010 ▸) (Fig. 7 ▸).

**Table 3:** Experimental details

Crystal data	
Chemical formula	C_20_H_31_N_3_S
*M*_r_	345.54
Crystal system, space group	Monoclinic, *P*2_1_/*c*
Temperature (K)	100
*a*, *b*, *c* (Å)	5.867 (3), 11.790 (4), 27.983 (9)
β (°)	94.907 (14)
*V* (Å^3^)	1928.7 (12)
*Z*	4
Radiation type	Mo *K*α
μ (mm^−1^)	0.17
Crystal size (mm)	0.21 × 0.20 × 0.15

Data collection	
Diffractometer	Bruker D8 Venture Photon 100 area detector diffractometer
Absorption correction	Multi-scan (*SADABS*; Krause *et al.*, 2015 ▸)
*T*_min_, *T*_max_	0.690, 0.746
No. of measured, independent and observed [*I* > 2σ(*I*)] reflections	31284, 4822, 3250
*R*_int_	0.092
(sin θ/λ)_max_ (Å^−1^)	0.668

Refinement	
*R*[*F* ^2^ > 2σ(*F* ^2^)], *wR*(*F* ^2^), *S*	0.079, 0.201, 1.06
No. of reflections	4822
No. of parameters	243
H-atom treatment	H-atom parameters constrained
Δρ_max_, Δρ_min_ (e Å^−3^)	0.70, −0.43

Computer programs: *APEX3* and *SAINT* (Bruker, 2015 ▸), *SHELXT2014/5* (Sheldrick, 2015*a* ▸), *SHELXL2018/3* (Sheldrick, 2015*b* ▸), *DIAMOND* (Brandenburg, 2006 ▸), CrystalExplorer (Wolff *et al.*, 2012 ▸), *WinGX* (Farrugia, 2012 ▸), *publCIF* (Westrip, 2010 ▸) and *enCIFer* (Allen *et al.*, 2004 ▸).

## full crystallographic data

### Crystal data

**Table:**

C_20_H_31_N_3_S	*F*(000) = 752
*M**_r_* = 345.54	*D*_x_ = 1.190 Mg m^−^^3^
Monoclinic, *P*2_1_/*c*	Mo *K*α radiation, λ = 0.71073 Å
*a* = 5.867 (3) Å	Cell parameters from 9284 reflections
*b* = 11.790 (4) Å	θ = 2.3–28.0°
*c* = 27.983 (9) Å	µ = 0.17 mm^−^^1^
β = 94.907 (14)°	*T* = 100 K
*V* = 1928.7 (12) Å^3^	Block, colorless
*Z* = 4	0.21 × 0.20 × 0.15 mm

### Data collection

**Table:**

Bruker D8 Venture Photon 100 area detector diffractometer	3250 reflections with *I* > 2σ(*I*)
Radiation source: microfocus X-ray tube, Bruker D8 Venture	*R*_int_ = 0.092
φ and ω scans	θ_max_ = 28.4°, θ_min_ = 2.3°
Absorption correction: multi-scan (SADABS; Krause *et al.*, 2015)	*h* = −7→7
*T*_min_ = 0.690, *T*_max_ = 0.746	*k* = −15→15
31284 measured reflections	*l* = −37→34
4822 independent reflections	

### Refinement

**Table:**

Refinement on *F*^2^	Primary atom site location: structure-invariant direct methods
Least-squares matrix: full	Hydrogen site location: inferred from neighbouring sites
*R*[*F*^2^ > 2σ(*F*^2^)] = 0.079	H-atom parameters constrained
*wR*(*F*^2^) = 0.201	*w* = 1/[σ^2^(*F*_o_^2^) + (0.0692*P*)^2^ + 4.1581*P*] where *P* = (*F*_o_^2^ + 2*F*_c_^2^)/3
*S* = 1.06	(Δ/σ)_max_ < 0.001
4822 reflections	Δρ_max_ = 0.70 e Å^−^^3^
243 parameters	Δρ_min_ = −0.43 e Å^−^^3^
0 restraints	

### Special details

**Table:**

Geometry. All esds (except the esd in the dihedral angle between two l.s. planes) are estimated using the full covariance matrix. The cell esds are taken into account individually in the estimation of esds in distances, angles and torsion angles; correlations between esds in cell parameters are only used when they are defined by crystal symmetry. An approximate (isotropic) treatment of cell esds is used for estimating esds involving l.s. planes.

### Fractional atomic coordinates and isotropic or equivalent isotropic displacement parameters (Å^2^)

**Table:**

	*x*	*y*	*z*	*U*_iso_*/*U*_eq_	Occ. (<1)
C1	0.4228 (6)	0.8742 (3)	0.34890 (11)	0.0297 (7)	
H1A	0.317178	0.837602	0.325720	0.044*	
H1B	0.572363	0.874803	0.337439	0.044*	
H1C	0.373714	0.950678	0.353735	0.044*	
C2	0.2556 (5)	0.8079 (2)	0.42119 (10)	0.0201 (6)	
C3	0.5694 (5)	0.6639 (2)	0.51451 (10)	0.0189 (6)	
C4	0.7938 (5)	0.6024 (3)	0.52273 (11)	0.0246 (7)	
H4A	0.767666	0.527218	0.534209	0.037*	
H4B	0.892159	0.642732	0.546098	0.037*	
H4C	0.864955	0.598124	0.493142	0.037*	
C5	0.4408 (5)	0.6874 (2)	0.55703 (10)	0.0174 (6)	
C6	0.3814 (5)	0.5991 (2)	0.58617 (10)	0.0173 (6)	
H6	0.420807	0.525634	0.578021	0.021*	
C7	0.2647 (5)	0.6151 (2)	0.62739 (10)	0.0161 (6)	
C8	0.2060 (5)	0.5121 (2)	0.65701 (10)	0.0198 (6)	
C9A	0.1338 (12)	0.5514 (4)	0.7065 (2)	0.0204 (15)	0.667 (13)
H9A1	0.067164	0.487592	0.722261	0.025*	0.667 (13)
H9A2	0.269015	0.575048	0.726400	0.025*	0.667 (13)
C10A	−0.0367 (13)	0.6481 (4)	0.7029 (2)	0.0188 (13)	0.667 (13)
H10A	−0.165910	0.626313	0.680229	0.023*	0.667 (13)
C9B	0.018 (2)	0.5468 (8)	0.6880 (4)	0.022 (3)	0.333 (13)
H9B1	−0.011379	0.484838	0.709389	0.026*	0.333 (13)
H9B2	−0.121129	0.560822	0.667565	0.026*	0.333 (13)
C10B	0.079 (3)	0.6527 (9)	0.7176 (4)	0.021 (3)	0.333 (13)
H10B	0.227432	0.644511	0.736163	0.025*	0.333 (13)
C18	−0.1252 (7)	0.6661 (3)	0.75207 (12)	0.0351 (9)	
H18A	−0.233592	0.727213	0.750333	0.053*	0.667 (13)
H18B	−0.198066	0.597947	0.761783	0.053*	0.667 (13)
H18C	0.000513	0.684304	0.775024	0.053*	0.667 (13)
H18D	−0.098157	0.731769	0.772011	0.053*	0.333 (13)
H18E	−0.267837	0.674553	0.732900	0.053*	0.333 (13)
H18F	−0.131339	0.599834	0.771876	0.053*	0.333 (13)
C11	0.0770 (5)	0.7569 (2)	0.68317 (10)	0.0206 (6)	
C12	0.2048 (5)	0.7260 (2)	0.63909 (10)	0.0181 (6)	
C13	0.2671 (5)	0.8150 (2)	0.60974 (10)	0.0211 (6)	
H13	0.226173	0.888377	0.617683	0.025*	
C14	0.3863 (5)	0.7999 (2)	0.56956 (11)	0.0208 (6)	
C15	0.4612 (6)	0.9020 (3)	0.54276 (12)	0.0299 (7)	
H15A	0.574184	0.879635	0.521721	0.045*	
H15B	0.525624	0.957293	0.565178	0.045*	
H15C	0.331752	0.934086	0.524249	0.045*	
C16	0.4184 (6)	0.4431 (3)	0.67218 (16)	0.0464 (11)	
H16A	0.484691	0.415578	0.644208	0.070*	
H16B	0.377618	0.380093	0.691427	0.070*	
H16C	0.527303	0.490084	0.690511	0.070*	
C17	0.0399 (8)	0.4351 (3)	0.62847 (14)	0.0493 (11)	
H17A	−0.096516	0.476586	0.618334	0.074*	
H17B	0.001698	0.372212	0.648087	0.074*	
H17C	0.108789	0.407383	0.600831	0.074*	
C19	0.2376 (7)	0.8235 (4)	0.71795 (14)	0.0469 (11)	
H19A	0.160960	0.842218	0.745876	0.070*	
H19B	0.282993	0.891975	0.702797	0.070*	
H19C	0.370519	0.778582	0.727203	0.070*	
C20	−0.1305 (6)	0.8300 (4)	0.66802 (14)	0.0421 (10)	
H20A	−0.235503	0.787662	0.646723	0.063*	
H20B	−0.082284	0.896632	0.651908	0.063*	
H20C	−0.204601	0.851976	0.695883	0.063*	
N1	0.4303 (4)	0.8132 (2)	0.39367 (9)	0.0217 (5)	
H1	0.554520	0.778200	0.403201	0.026*	
N2	0.2951 (4)	0.7419 (2)	0.46157 (9)	0.0215 (5)	
H2	0.190095	0.733073	0.480892	0.026*	
N3	0.5059 (4)	0.6897 (2)	0.47075 (9)	0.0196 (5)	
S1	0.00559 (14)	0.87428 (7)	0.41079 (3)	0.0293 (2)	

### Atomic displacement parameters (Å^2^)

**Table:**

	*U*^11^	*U*^22^	*U*^33^	*U*^12^	*U*^13^	*U*^23^
C1	0.0322 (18)	0.0341 (18)	0.0226 (15)	0.0026 (15)	0.0019 (13)	0.0073 (14)
C2	0.0222 (15)	0.0177 (14)	0.0199 (14)	−0.0027 (11)	−0.0006 (12)	−0.0013 (11)
C3	0.0199 (15)	0.0181 (13)	0.0190 (14)	−0.0023 (11)	0.0039 (11)	0.0011 (11)
C4	0.0213 (16)	0.0292 (16)	0.0237 (15)	0.0049 (12)	0.0040 (12)	0.0034 (12)
C5	0.0164 (14)	0.0200 (14)	0.0161 (13)	−0.0018 (11)	0.0022 (11)	−0.0005 (11)
C6	0.0192 (15)	0.0127 (12)	0.0203 (14)	0.0017 (10)	0.0028 (11)	0.0001 (10)
C7	0.0161 (14)	0.0136 (12)	0.0182 (13)	0.0002 (10)	−0.0006 (11)	0.0024 (10)
C8	0.0246 (16)	0.0156 (13)	0.0198 (14)	0.0001 (11)	0.0057 (12)	0.0013 (11)
C9A	0.029 (3)	0.016 (2)	0.016 (3)	−0.002 (2)	0.005 (2)	−0.0001 (18)
C10A	0.021 (3)	0.023 (2)	0.013 (2)	0.005 (2)	0.002 (2)	−0.0004 (18)
C9B	0.026 (7)	0.024 (5)	0.015 (5)	−0.003 (4)	0.001 (5)	−0.004 (4)
C10B	0.019 (6)	0.033 (5)	0.011 (5)	0.007 (4)	0.001 (4)	0.003 (4)
C18	0.052 (2)	0.0295 (17)	0.0265 (17)	0.0067 (16)	0.0211 (16)	0.0024 (14)
C11	0.0246 (16)	0.0169 (13)	0.0207 (14)	0.0022 (12)	0.0040 (12)	−0.0011 (11)
C12	0.0205 (15)	0.0160 (13)	0.0174 (14)	−0.0003 (11)	−0.0013 (11)	−0.0019 (10)
C13	0.0270 (16)	0.0141 (13)	0.0223 (15)	0.0009 (11)	0.0020 (12)	0.0004 (11)
C14	0.0233 (16)	0.0173 (13)	0.0218 (15)	−0.0020 (12)	0.0028 (12)	0.0014 (11)
C15	0.045 (2)	0.0150 (14)	0.0304 (17)	−0.0027 (13)	0.0087 (15)	0.0030 (12)
C16	0.033 (2)	0.037 (2)	0.069 (3)	−0.0020 (16)	−0.0022 (19)	0.035 (2)
C17	0.063 (3)	0.041 (2)	0.040 (2)	−0.033 (2)	−0.0184 (19)	0.0190 (18)
C19	0.030 (2)	0.070 (3)	0.041 (2)	−0.0010 (19)	0.0093 (17)	−0.035 (2)
C20	0.029 (2)	0.067 (3)	0.0313 (19)	0.0194 (18)	0.0073 (15)	0.0040 (18)
N1	0.0247 (14)	0.0228 (13)	0.0179 (12)	0.0035 (10)	0.0030 (10)	0.0038 (10)
N2	0.0180 (13)	0.0256 (13)	0.0214 (13)	0.0030 (10)	0.0038 (10)	0.0040 (10)
N3	0.0180 (13)	0.0214 (12)	0.0193 (12)	0.0002 (10)	0.0009 (10)	0.0022 (10)
S1	0.0228 (4)	0.0270 (4)	0.0376 (5)	0.0034 (3)	0.0002 (3)	0.0079 (3)

### Geometric parameters (Å, º)

**Table:**

C1—N1	1.442 (4)	C10B—C18	1.609 (11)
C1—H1A	0.9600	C10B—H10B	0.9800
C1—H1B	0.9600	C18—H18A	0.9600
C1—H1C	0.9600	C18—H18B	0.9600
C2—N1	1.335 (4)	C18—H18C	0.9600
C2—N2	1.376 (4)	C18—H18D	0.9600
C2—S1	1.666 (3)	C18—H18E	0.9600
C3—N3	1.286 (4)	C18—H18F	0.9600
C3—C5	1.489 (4)	C11—C19	1.515 (5)
C3—C4	1.503 (4)	C11—C20	1.523 (5)
C4—H4A	0.9600	C11—C12	1.541 (4)
C4—H4B	0.9600	C12—C13	1.400 (4)
C4—H4C	0.9600	C13—C14	1.386 (4)
C5—C6	1.385 (4)	C13—H13	0.9300
C5—C14	1.416 (4)	C14—C15	1.503 (4)
C6—C7	1.403 (4)	C15—H15A	0.9600
C6—H6	0.9300	C15—H15B	0.9600
C7—C12	1.400 (4)	C15—H15C	0.9600
C7—C8	1.526 (4)	C16—H16A	0.9600
C8—C17	1.510 (5)	C16—H16B	0.9600
C8—C9B	1.515 (10)	C16—H16C	0.9600
C8—C16	1.518 (5)	C17—H17A	0.9600
C8—C9A	1.554 (5)	C17—H17B	0.9600
C9A—C10A	1.515 (9)	C17—H17C	0.9600
C9A—H9A1	0.9700	C19—H19A	0.9600
C9A—H9A2	0.9700	C19—H19B	0.9600
C10A—C18	1.527 (5)	C19—H19C	0.9600
C10A—C11	1.568 (6)	C20—H20A	0.9600
C10A—H10A	0.9800	C20—H20B	0.9600
C9B—C10B	1.524 (19)	C20—H20C	0.9600
C9B—H9B1	0.9700	N1—H1	0.8600
C9B—H9B2	0.9700	N2—N3	1.386 (3)
C10B—C11	1.560 (10)	N2—H2	0.8600
			
N1—C1—H1A	109.5	H18A—C18—H18C	109.5
N1—C1—H1B	109.5	H18B—C18—H18C	109.5
H1A—C1—H1B	109.5	C10B—C18—H18D	109.5
N1—C1—H1C	109.5	C10B—C18—H18E	109.5
H1A—C1—H1C	109.5	H18D—C18—H18E	109.5
H1B—C1—H1C	109.5	C10B—C18—H18F	109.5
N1—C2—N2	114.6 (3)	H18D—C18—H18F	109.5
N1—C2—S1	125.8 (2)	H18E—C18—H18F	109.5
N2—C2—S1	119.6 (2)	C19—C11—C20	108.9 (3)
N3—C3—C5	126.3 (3)	C19—C11—C12	108.6 (3)
N3—C3—C4	116.0 (3)	C20—C11—C12	110.1 (3)
C5—C3—C4	117.7 (2)	C19—C11—C10B	92.3 (6)
C3—C4—H4A	109.5	C20—C11—C10B	125.6 (6)
C3—C4—H4B	109.5	C12—C11—C10B	109.2 (4)
H4A—C4—H4B	109.5	C19—C11—C10A	117.3 (4)
C3—C4—H4C	109.5	C20—C11—C10A	102.0 (4)
H4A—C4—H4C	109.5	C12—C11—C10A	109.8 (3)
H4B—C4—H4C	109.5	C7—C12—C13	118.6 (3)
C6—C5—C14	119.0 (2)	C7—C12—C11	123.9 (2)
C6—C5—C3	120.0 (2)	C13—C12—C11	117.5 (2)
C14—C5—C3	120.9 (2)	C14—C13—C12	123.7 (3)
C5—C6—C7	123.2 (3)	C14—C13—H13	118.1
C5—C6—H6	118.4	C12—C13—H13	118.1
C7—C6—H6	118.4	C13—C14—C5	117.5 (3)
C12—C7—C6	117.9 (2)	C13—C14—C15	119.5 (3)
C12—C7—C8	122.9 (2)	C5—C14—C15	123.0 (3)
C6—C7—C8	119.2 (2)	C14—C15—H15A	109.5
C17—C8—C9B	89.8 (6)	C14—C15—H15B	109.5
C17—C8—C16	107.6 (3)	H15A—C15—H15B	109.5
C9B—C8—C16	127.2 (6)	C14—C15—H15C	109.5
C17—C8—C7	110.9 (3)	H15A—C15—H15C	109.5
C9B—C8—C7	107.7 (4)	H15B—C15—H15C	109.5
C16—C8—C7	111.1 (3)	C8—C16—H16A	109.5
C17—C8—C9A	116.0 (4)	C8—C16—H16B	109.5
C16—C8—C9A	101.0 (4)	H16A—C16—H16B	109.5
C7—C8—C9A	109.8 (3)	C8—C16—H16C	109.5
C10A—C9A—C8	113.1 (5)	H16A—C16—H16C	109.5
C10A—C9A—H9A1	109.0	H16B—C16—H16C	109.5
C8—C9A—H9A1	109.0	C8—C17—H17A	109.5
C10A—C9A—H9A2	109.0	C8—C17—H17B	109.5
C8—C9A—H9A2	109.0	H17A—C17—H17B	109.5
H9A1—C9A—H9A2	107.8	C8—C17—H17C	109.5
C9A—C10A—C18	108.5 (5)	H17A—C17—H17C	109.5
C9A—C10A—C11	110.0 (5)	H17B—C17—H17C	109.5
C18—C10A—C11	113.1 (4)	C11—C19—H19A	109.5
C9A—C10A—H10A	108.4	C11—C19—H19B	109.5
C18—C10A—H10A	108.4	H19A—C19—H19B	109.5
C11—C10A—H10A	108.4	C11—C19—H19C	109.5
C8—C9B—C10B	112.6 (11)	H19A—C19—H19C	109.5
C8—C9B—H9B1	109.1	H19B—C19—H19C	109.5
C10B—C9B—H9B1	109.1	C11—C20—H20A	109.5
C8—C9B—H9B2	109.1	C11—C20—H20B	109.5
C10B—C9B—H9B2	109.1	H20A—C20—H20B	109.5
H9B1—C9B—H9B2	107.8	C11—C20—H20C	109.5
C9B—C10B—C11	108.7 (10)	H20A—C20—H20C	109.5
C9B—C10B—C18	104.7 (10)	H20B—C20—H20C	109.5
C11—C10B—C18	109.0 (7)	C2—N1—C1	123.8 (3)
C9B—C10B—H10B	111.4	C2—N1—H1	118.1
C11—C10B—H10B	111.4	C1—N1—H1	118.1
C18—C10B—H10B	111.4	C2—N2—N3	119.3 (2)
C10A—C18—H18A	109.5	C2—N2—H2	120.4
C10A—C18—H18B	109.5	N3—N2—H2	120.4
H18A—C18—H18B	109.5	C3—N3—N2	117.6 (2)
C10A—C18—H18C	109.5		
			
N3—C3—C5—C6	122.5 (3)	C18—C10A—C11—C19	43.8 (6)
C4—C3—C5—C6	−56.4 (4)	C9A—C10A—C11—C20	163.7 (5)
N3—C3—C5—C14	−60.3 (4)	C18—C10A—C11—C20	−74.9 (5)
C4—C3—C5—C14	120.7 (3)	C9A—C10A—C11—C12	47.0 (6)
C14—C5—C6—C7	1.4 (4)	C18—C10A—C11—C12	168.4 (4)
C3—C5—C6—C7	178.6 (3)	C6—C7—C12—C13	−1.0 (4)
C5—C6—C7—C12	0.5 (4)	C8—C7—C12—C13	179.8 (3)
C5—C6—C7—C8	179.7 (3)	C6—C7—C12—C11	−179.9 (3)
C12—C7—C8—C17	114.7 (4)	C8—C7—C12—C11	0.9 (4)
C6—C7—C8—C17	−64.4 (4)	C19—C11—C12—C7	112.6 (3)
C12—C7—C8—C9B	17.9 (7)	C20—C11—C12—C7	−128.3 (3)
C6—C7—C8—C9B	−161.3 (7)	C10B—C11—C12—C7	13.2 (7)
C12—C7—C8—C16	−125.6 (3)	C10A—C11—C12—C7	−16.9 (5)
C6—C7—C8—C16	55.2 (4)	C19—C11—C12—C13	−66.3 (4)
C12—C7—C8—C9A	−14.8 (5)	C20—C11—C12—C13	52.8 (4)
C6—C7—C8—C9A	166.0 (4)	C10B—C11—C12—C13	−165.6 (7)
C17—C8—C9A—C10A	−79.7 (6)	C10A—C11—C12—C13	164.3 (4)
C16—C8—C9A—C10A	164.3 (5)	C7—C12—C13—C14	−0.2 (5)
C7—C8—C9A—C10A	47.0 (7)	C11—C12—C13—C14	178.7 (3)
C8—C9A—C10A—C18	170.6 (4)	C12—C13—C14—C5	2.0 (5)
C8—C9A—C10A—C11	−65.3 (7)	C12—C13—C14—C15	−175.1 (3)
C17—C8—C9B—C10B	−165.1 (10)	C6—C5—C14—C13	−2.5 (4)
C16—C8—C9B—C10B	82.6 (10)	C3—C5—C14—C13	−179.7 (3)
C7—C8—C9B—C10B	−53.2 (12)	C6—C5—C14—C15	174.5 (3)
C8—C9B—C10B—C11	70.2 (14)	C3—C5—C14—C15	−2.6 (5)
C8—C9B—C10B—C18	−173.3 (6)	N2—C2—N1—C1	177.7 (3)
C9B—C10B—C11—C19	−156.1 (10)	S1—C2—N1—C1	−3.1 (4)
C18—C10B—C11—C19	90.3 (8)	N1—C2—N2—N3	1.2 (4)
C9B—C10B—C11—C20	88.6 (9)	S1—C2—N2—N3	−178.1 (2)
C18—C10B—C11—C20	−25.0 (12)	C5—C3—N3—N2	−1.8 (4)
C9B—C10B—C11—C12	−45.5 (12)	C4—C3—N3—N2	177.2 (2)
C18—C10B—C11—C12	−159.1 (6)	C2—N2—N3—C3	155.7 (3)
C9A—C10A—C11—C19	−77.6 (5)		

### Hydrogen-bond geometry (Å, º)

**Table:**

*D*—H···*A*	*D*—H	H···*A*	*D*···*A*	*D*—H···*A*
N1—H1···S1^i^	0.86	2.87	3.445 (3)	126

Symmetry code: (i) *x*+1, *y*, *z*.
